# Supplementary material for: Colon mucosal proteomics of ankylosing spondylitis versus gut inflammation
Source: PLoS One. 2024 Dec 13;19(12):e0315324. doi: 10.1371/journal.pone.0315324 (PMC11642932; doi:10.1371/journal.pone.0315324)

Supplementary Figure 1. Principal Component Analysis

This Principal Component Analysis (PCA) plot represents the proteomic profiles of colonic mucosal tissues across four groups: healthy individuals (Group A), patients with gut inflammation only (Group B), patients with AS only (Group C), and patients with AS combined with gut inflammation (Group D). PC1 and PC2 explain 0.28 and 0.12 of the total variance, respectively. The clear separation between the groups along the principal components indicates significant differences in proteomic profiles.

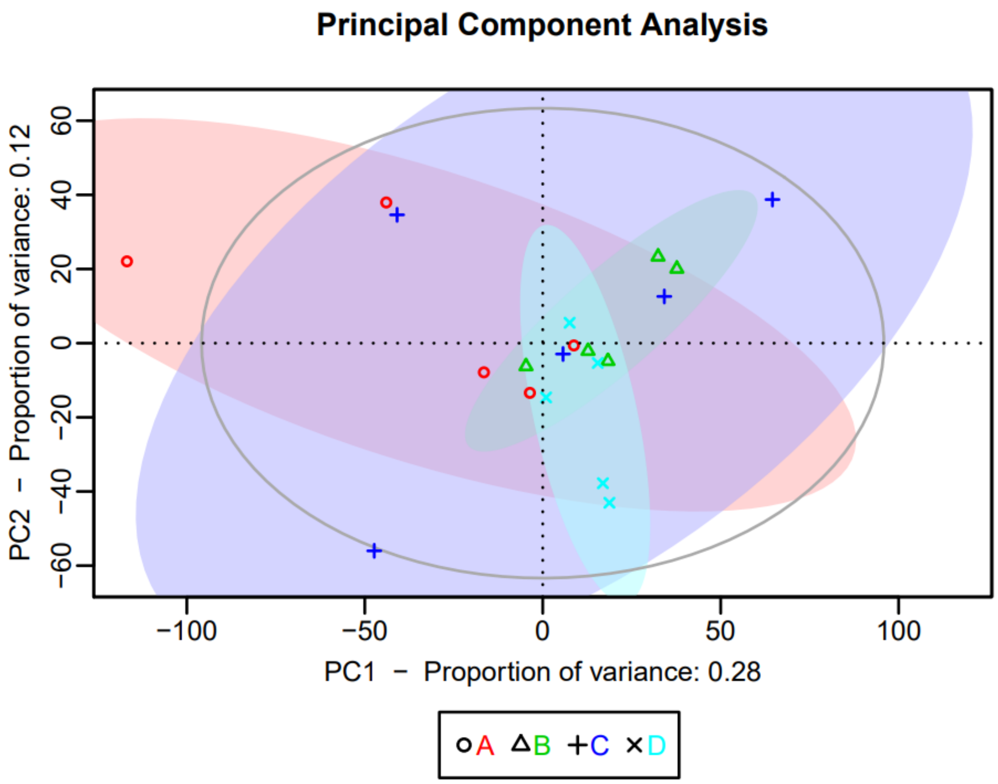

Supplement: S1 Fig — This Principal Component Analysis (PCA) plot represents the proteomic profiles of colonic mucosal tissues across four groups: healthy individuals (Group A), patients with gut inflammation only (Group B), patients with AS only (Group C), and patients with AS combined with gut inflammation (Group D). PC1 and PC2 explain 0.28 and 0.12 of the total variance, respectively. The clear separation between the groups along the principal components indicates significant differences in proteomic profiles. (PDF) [file pone.0315324.s001.pdf]
